# Excellent anti-mildew effect of essential oil impregnation on sliced veneer plybamboo and its anti-mildew mechanism

**DOI:** 10.1128/spectrum.01652-24

**Published:** 2024-10-10

**Authors:** Qichao Bao, Yingying Shan, Fei Yang, Jiawei Zhu, Chungui Du, Yuting Wang, Yuran Shao, Chunlin Liu, Shiqin Chen, Ying Ran, Wenxiu Yin

**Affiliations:** 1 Bamboo Industry Institute, Zhejiang A&F University, Hangzhou, China; 2 College of Chemistry and Materials Engineering, Zhejiang A&F University, Hangzhou, China; Southwest University, Chongqing, China

**Keywords:** antimildew, spices essential oil, slice veneer plybamboo, natural antimildew agents, Oxford cup method

## Abstract

Sliced bamboo veneer used as a high-end decoration material is a highly innovative material for the deep processing of bamboo. However, bamboo is rich in starch and small molecular soluble sugars, making it susceptible to mildew infection and limiting the wide application of sliced veneer plybamboo. Spice essential oils are considered green and safe antimildew agents, which are cheap and accessible. The natural phenolic substances in plant essential oil have a good inhibitory effect on bamboo mildew. In this study, three types of spice essential oils (clove essential oil, oregano essential oil, and fennel essential oil) were employed, and their antifungal activity against bamboo mildews was assessed using the Oxford cup method, scanning electron microscopy, transmission electron microscopy, and a micro pH meter. The results demonstrated that the diameters of the Inhibition Zone against four common bamboo mildews (AN*, Aspergillus niger;* TV*, Trichoderma viride;* PC, *Penicillium citrinum*; MM, Mixed Mildews) caused by clove essential oil were 25.68 mm, 23.22 mm, 30.68 mm, and 25.43 mm, respectively. As an explanation, clove essential oil can inhibit or eliminate mildew by damaging and disrupting the cell membrane of the bamboo mildew, leading to significant shrinkage, distortion, surface roughness, formation of holes, or partial structural cracks in the mildew’s mycelium. Additionally, it may interfere with and disrupt the pH balance of the intracellular and extracellular fluids within the cell. Furthermore, we also report that sliced veneer plybamboo impregnated with clove essential oil on each layer showed fine inhibition rates of 50%, 75%, 100%, and 25% against AN, TV, PC, and MM, respectively. This research underscores a sustainable approach to mildew prevention, crucial for advancing bamboo’s utilization in high-value furniture decor applications.

**IMPORTANCE:**

Mildew growth in sliced veneer plybamboo poses a significant challenge, particularly in its use for high-end furniture and decor. Traditionally, chemical treatments have been the primary solution though they often raise environmental concerns. Essential oils, with their well-documented antimicrobial properties, have emerged as an important natural and eco-friendly alternative for preventing mildew. These oils inhibit mildew growth effectively while offering a sustainable, non-toxic solution that reduces harm to both the environment and human health. By leveraging essential oils, it becomes possible to extend the lifespan of bamboo products, making them more durable and suitable for broader applications in furniture and decor, all while addressing the ecological limitations of conventional mildew prevention methods.

## INTRODUCTION

Bamboo plays multiple roles in the field of decoration: its natural esthetic characteristics give decoration unique texture and style and environmental protection, and the continuous innovation of processing technology for its application provides more possibilities, thus becoming the pursuit of environmental protection, high quality and innovative decorative materials (1, 2). Bamboo’s attributes, including rapid growth, eco-friendliness, and sustainability, make it an attractive choice for construction (3), furniture (4), decoration, and more (5). Sliced bamboo veneer is a new type of material for the deep processing of bamboo resources in China, which is made of bamboo processed into bamboo slices, glued into bamboo squares through a series of processes, and then softened and sliced into thin bamboo veneer materials (6, 7). Sliced bamboo veneer is currently used as a material for furniture and interior decoration (8), and it has produced significant economic benefits. However, compared to wood, bamboo contains a substantial amount of nutrients like soluble small-molecule polysaccharides, proteins, and starch, rendering it more susceptible to bacteria and mildew (9). This susceptibility not only impacts bamboo’s quality and lifespan but also poses potential health and safety risks. Although conventional chemical mildew inhibitors are effective, their high toxicity and residual levels pose severe environmental and human health risks. Hence, the search for efficient, environmentally friendly mildew inhibitors is imperative for the future utilization of bamboo materials. Currently, bamboo mildew treatment primarily involves physical and chemical methods (10). Given the limited effectiveness of physical methods, chemical approaches have taken precedence. However, the use of chemical mildew inhibitors like sulfur, pentachlorophenol, and copper-chromium-arsenic has raised concerns due to their adverse effects on the environment (11) and human health. Consequently, the future direction in bamboo mildew control focuses on the development of eco-friendly mildew inhibitors or natural anti-mildew agents.

In this context, our study aims to investigate a novel approach to prevent bamboo mildew, specifically through the utilization of essential oils derived from spices. Aromatic essential oils, being natural extracts, are known for their potent antimicrobial properties due to the concentrated natural bioactive compounds they contain (12). These essential oils find extensive use in various domains, including food (13), healthcare (14), medicine, cosmetics, and more. Nevertheless, there is currently limited research on the utilization of spice essential oils for controlling bamboo mildew. The primary goal of our research is to assess the potential of essential oils in the realm of bamboo mildew prevention and to explore innovative and feasible applications, particularly in the context of sliced veneer plybamboo, a significant form of bamboo.

This study aims to determine the effectiveness of aromatic spice essential oils in preventing mildew contamination in thinly sliced bamboo veneer and compares it with traditional chemical anti-mildew agents. It also seeks to uncover the mechanisms behind the mildew-prevention properties of spice essential oils. This research proposes an eco-friendly and sustainable method for preventing mildew in bamboo, potentially bringing significant improvements to the bamboo industry. Moreover, it contributes to the broader utilization of natural plant extracts in bamboo preservation, To promote the application of bamboo in the field of furniture and building materials.

## MATERIALS AND METHODS

### Materials preparation

Clove essential oil (99%), oregano essential oil (99%), and fennel essential oil (99%) were purchased from Guangzhou Hongli Biotechnology Co., Ltd, China. Tween 80, chemically pure, was purchased from Shanghai Lingfeng Chemical Reagent Co., Ltd, China. Sodium dihydrogen phosphate and disodium hydrogen phosphate, both analytical pure, were purchased from Sinopharm Chemical Reagent Co., Ltd, China.

Sliced bamboo veneers with sizes of approximately 2,000 mm (length) × 450 mm (width) × 0.55 mm (thickness) were purchased from Hangzhou Senrui Bamboo & Wood Industry Co. LTD. Modified Urea-formaldehyde (UF) Resin was procured from Hangzhou Senrui Wood Industry Co., Ltd, China.

Sodium dihydrogen phosphate and disodium hydrogen phosphate, analytical pure, were purchased from Sinopharm Chemical Reagent Co., Ltd, Shanghai, China. AN (*Aspergillus niger*), TV (*Trichoderma viride*), PC (*Penicillium citrinum*), and mixed mildews (MM, which was equal proportions of AN, TV, and PC) were the experimental strains used. It was isolated from naturally occurring bambusa mildew by microbiology research group of Zhejiang A&F University.

### Methods

#### Preparation of Potato Dextrose Agar

Boil 200 g of peeled potatoes in deionized water for 20 min and then filter. Add 20 g of glucose, 24 g of agar, and enough water to make 1,000 mL. Stir until dissolved and then sterilize at 121°C for 2.5 h to prepare the PDA medium for fungal culture.

#### Preparation of mildew suspensions

The flasks containing appropriate amounts of sterile water and small glass beads were sterilized in an autoclave at 121°C and 0.1 MPa pressure for 2.5 h. Following this, under super clean bench sterile conditions, mycelia and spores of tested strains were added to a sterilized wide-mouth flask using an inoculating needle, and the flask was shaken for 10–15 min to fully mix and obtain a homogeneous mildew suspension for inoculation.

#### Preparation of spice essential oil solutions

A total of 20 g each of the high-purity oils was placed in their respective beakers, and then Tween 80, accounting for 2% of the total volume, and a small amount of deionized water was evenly added to every beaker. Afterward, the mixtures were moved into the respective volumetric flasks to fix the volume. Finally, the oil solutions with the concentration gradient of 3.125 mg/mL, 6.25 mg/mL, 12.5 mg/mL, 25 mg/mL, 50 mg/mL, and 100 mg/mL were prepared successfully.

#### Determining the anti-mildew activity of spice essential oils

Here, the Oxford Cup method (15) was used to determine the fungistatic activity of spice essential oils against bamboo mildew. First, 80 µL of bamboo mildew suspension was evenly coated on the PDA plate, and the sterilized Oxford Cup (8 mm outside diameter and 6 mm inside diameter) was placed on the coated PDA plate. Then, 80 µL of spice essential oil solutions of varying concentrations was injected into the Oxford Cup. The Petri dishes were sealed with sterile sealing films and placed at 4°C for diffusion for 2 h. Finally, they were incubated at 28°C and 85% ± 5% humidity. After 3 days of cultivation, the diameter of the anti-mildew zone was measured using a cross-over method and a vernier caliper. The diameter of the inhibition zone was measured by a vernier caliper after 3 days of incubation using the cross-over method. The inhibition zone diameter was evaluated according to the following criteria: a diameter >20 mm was considered extremely sensitive; diameters between 15 and 20 mm are highly sensitive; a diameter between 10 and 15 mm is moderately sensitive; a diameter <10 mm is low sensitivity; a diameter of approximately 0 mm is insensitive. The experiment was performed in triplicate for each concentration, and the average 140 value of the results was taken to calculate the fungistatic rate according to formula (1). Tween 80 was used as a control.


(1) $$B=\frac{D_{1}-D_{0}}{D_{1}}\times 100\%$$

In the formula, *B* is the Fungal inhibition rate; *D*_0_ is the diameter of the inhibition zone in the control group in millimeters (mm); *D*_1_ is the diameter of the inhibition zone in the treatment group in millimeters (mm)

#### Determining minimum inhibitory concentration and minimum fungicidal concentration

Based on the Oxford Cup test results of the three spice essential oil solutions, MIC and MFC of spice essential oil were determined by the multiple dilution method (16). Each oil solution was evenly added to the respective PDA plates at varying concentrations. Following this, 80 µL of bacterial suspension was evenly coated on the surface of the PDA plates, and the Petri dishes were sealed with sterile sealing films and finally incubated at 28°C and 85% ± 5% humidity. The growth of the mildew cultures was observed after 3 days, and the minimum concentration of the respective spice essential oils inhibiting the growth of cultures was used as MIC. Based on the MIC value, the culture was continued for 7 days, and the minimum concentration for the complete inhibition of the growth was determined as MFC. Tween 80 was used as a control.

#### Effect of spice essential oils on mycelial morphology of the bamboo mildew

The effect of three spice essential oils on the mycelial morphology of the bamboo mildew was observed by using SU8010 scanning electron microscope (SEM). The mildew cake with a diameter of 8.0 mm was removed from the mildew culture medium after culturing for 7 days and placed on the PDA plate with concentrations of 0, MIC, and MFC of spice essential oils. Finally, the mildew was cultured at 28°C and 85% ± 5% humidity, and after 4 days, the mildew cake was cut off for samples for SEM observation.

The samples were fixed overnight with 2.5% glutaraldehyde at 4°C and then rinsed with phosphate buffer solution (PBS, pH 7.0) three times for 15 min each. Then, the samples were dehydrated in an ethanol gradient (30%, 50%, 70%, 80%, 90%, and 95%) for 15 min each. Finally, the samples were dehydrated with the anhydrous ethanol for 20 min and were subjected to vacuum freeze-drying and gold spraying treatment. Following this, the mycelial surface morphology was observed under SEM.

#### The influence of spice essential oils on the ultrastructure of bamboo mycorrhizal cells

In order to further investigate the mechanism of clove essential oil with high anti-mildew activity against bamboo, the Hitachi HT7800 transmission electron microscope (Transmission Electron Microscope, TEM) was used to observe the microstructure of the mildew cells treated with clove essential oil.

#### Effect of spice essential oil on the pH of the extracellular fluid of bamboo mildew

A micro-pH meter (PHS-3C) was used to investigate the pH changes of the extracellular fluid of the mildew after the citral treatment. Mildew spores cultured for 7 days were selected and washed thrice in PBS (pH 7.0). Subsequently, the appropriate amount of spore suspension was treated with the spice essential oil solutions with concentrations of 0, MIC, and MFC for 0 min, 30 min, 60 min, and 120 min, respectively. Then, 5 mL of spore suspension was taken to measure the pH value of the extracellular fluid in triplicate, with Tween 80 used as a control.

#### Preparation of anti-mildew sliced veneer plybamboo treated with clove essential oil

The sliced bamboo veneers (25 mm × 25 mm) were impregnated and dried until the moisture contents were reduced to 8%–12%, and then, the three-layer structure of sliced veneer plybamboos was prepared through the processes of sizing, forming, and hot pressing. All three sliced bamboo veneers glued in the control group were not impregnated with treated clove essential oil (noted as P_0_). The anti-mildew sliced veneer plybamboos were prepared by two methods: first, the core layer of sliced bamboo veneer was not impregnated with clove essential oil (47.04 mg/mL, 3 h) (the upper and lower layers of sliced bamboo veneer were impregnated with clove essential oil, noted as P_2_; second, all three sliced bamboo veneers were impregnated with clove essential oil (noted as P_3_) (see Fig. 8B). The adhesive was made of environmentally friendly modified urea-formaldehyde resin glue, and the core board was aged for 10 min after gluing, and the hot pressing process was hot pressing temperature 100°C, unit pressure 1 MPa, and hot pressing time 1 min/mm board thickness.

#### Appearance quality of clove essential oil anti-mildew sliced veneer plybamboo

In order to investigate the effect of clove essential oil on the appearance quality of anti-mildew sliced veneer plybamboo, the determination was made according to the relevant requirements of the forestry industry standard “Sliced bamboo veneer” (LY/T 2222-2013).

#### The physical and chemical properties of anti-mildew sliced veneer plybamboo treated with clove essential oil

According to the national standard “Plywood for general use” (GB/T 9846-2015) requirements, the physical and chemical properties such as moisture content and gluing strength of anti-mildew sliced veneer plybamboos were investigated. The moisture content is calculated according to equation (2).


(2) $$H=\frac{m_{0}-m_{1}}{m_{1}}\times 100\%$$

Glue strength specimens are prepared in accordance with Class B specimens, and the glue strength is calculated in accordance with formula (3).


(3) $$X=\frac{P_{max}}{b\times l}\times 0.9$$

In the formula, *X* is the bonding strength of the specimen in MPa (megapascals); *P*_max_ is the maximum breaking load in Newton (N); *b* is the width of the shear surface of the specimen in millimeters (mm); *l* is the length of the shear surface of the specimen in millimeters (mm).

#### Anti-mildew properties of sliced veneer plybamboo treated by clove essential oil

The relevant provisions of the forestry industry standard “Standard method of evaluating the resistance of wood-based panels to mold” (LY/T 2230-2013) were used to test the mildew resistance of three types of sliced veneer plybamboo (P_0_, P_2_, P_3_). During the mildew test cycle lasting 28 days, the experimental procedure involved utilizing Table 1 as a grading table for assessing mildew growth on the surface of artificial board specimens. Observations and recordings were made every 7 days, followed by an analysis of the sliced veneer plybamboo using AN, TV, PC, and MM techniques. The goal of this experiment was to test the mildew resistance of three types of sliced veneer plybamboo (P_0_, P_2_, P_3_). The control effect of clove essential oil on mildew of sliced veneer plybamboo was calculated according to formula (4) and determine the quality of man-made board mildew classification.

**TABLE 1:** Classification of mildew growth on the surface of wood-based panel specimens

Grading value	Description of mildew growth	Mildew growth
0	No growth	No mycelium growth on the surface
1	Trace growth	A little mycelium on the surface of the specimen, but the infection area is ≤10%
2	Slight growth	Slight growth of mycelium, the surface of the specimen infected area >10% but ≤30%
3	Moderate growth	Moderate growth of mycelium, the surface of the specimen infected area >30% but ≤60%
4	Severe growth	Mycorrhizal serious growth, the surface of the specimen infected area >60%


(4) $$E=\left(1-\frac{D_{1}}{D_{0}}\right)\times 100\%$$

In the formula, *E* is the effectiveness of prevention and control; *D*_1_ is the average infection value of sliced bamboo tablets treated with the spices essential oils; *D*_0_ is the average infection value of sliced bamboo tablets in the control group.

## RESULTS AND DISCUSSION

### Analysis of fungistatic properties of three spices essential oils against bamboo mildew

**Fig 1:**
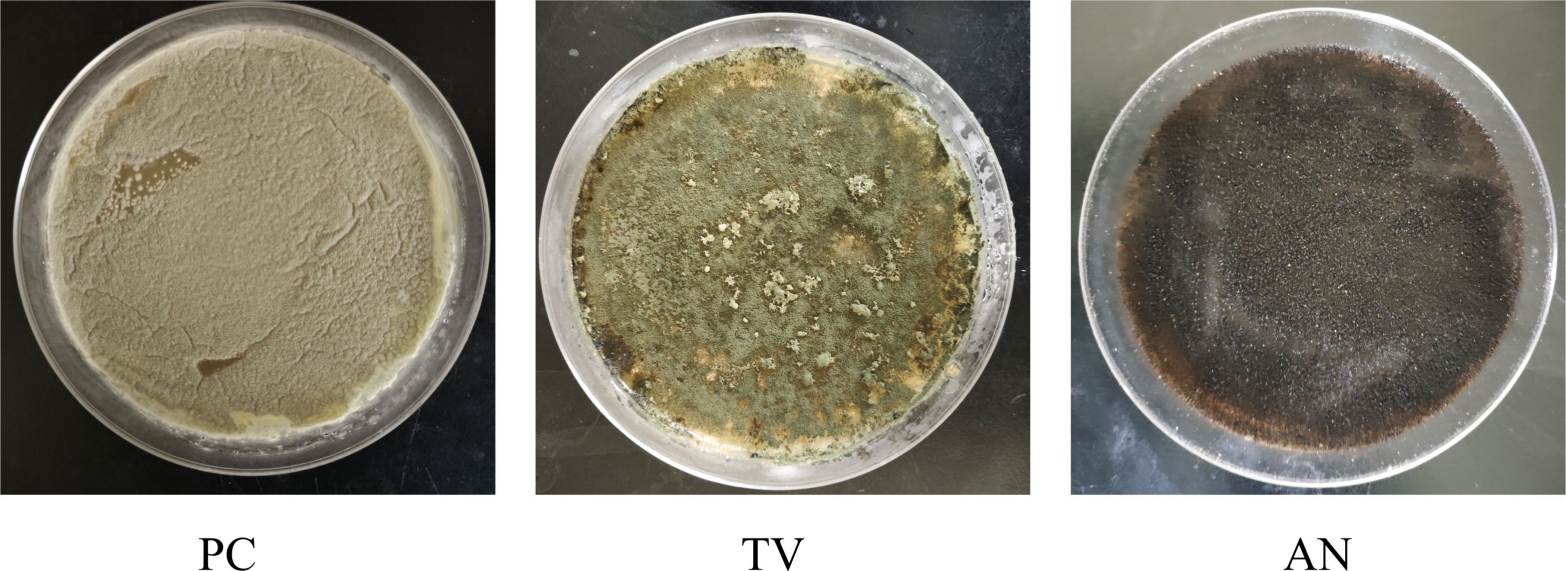
Colony images of three types of mildews.

The colonies of the three molds are shown in Fig. 1. The surface of *Penicillium citrinum* colonies is smooth with wrinkles and appears orange-yellow. *Trichoderma viride* colonies exhibit a bright green surface with some protrusions. *Aspergillus niger* starts as white during early growth but gradually turns black as it matures.

**Fig 2:**
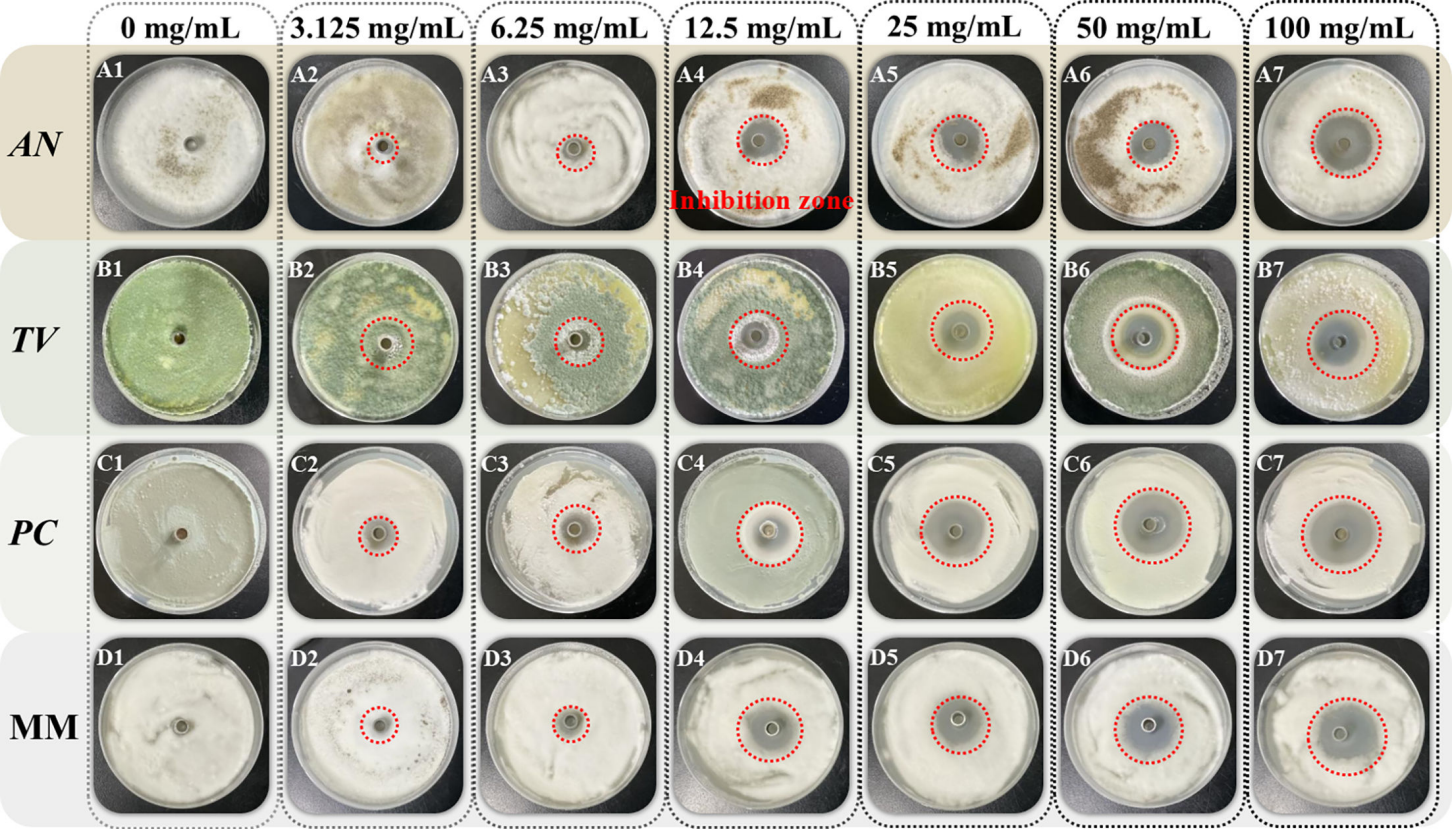
Inhibitory effect of clove essential oil on the growth of bamboo mildew.

**Fig 3:**
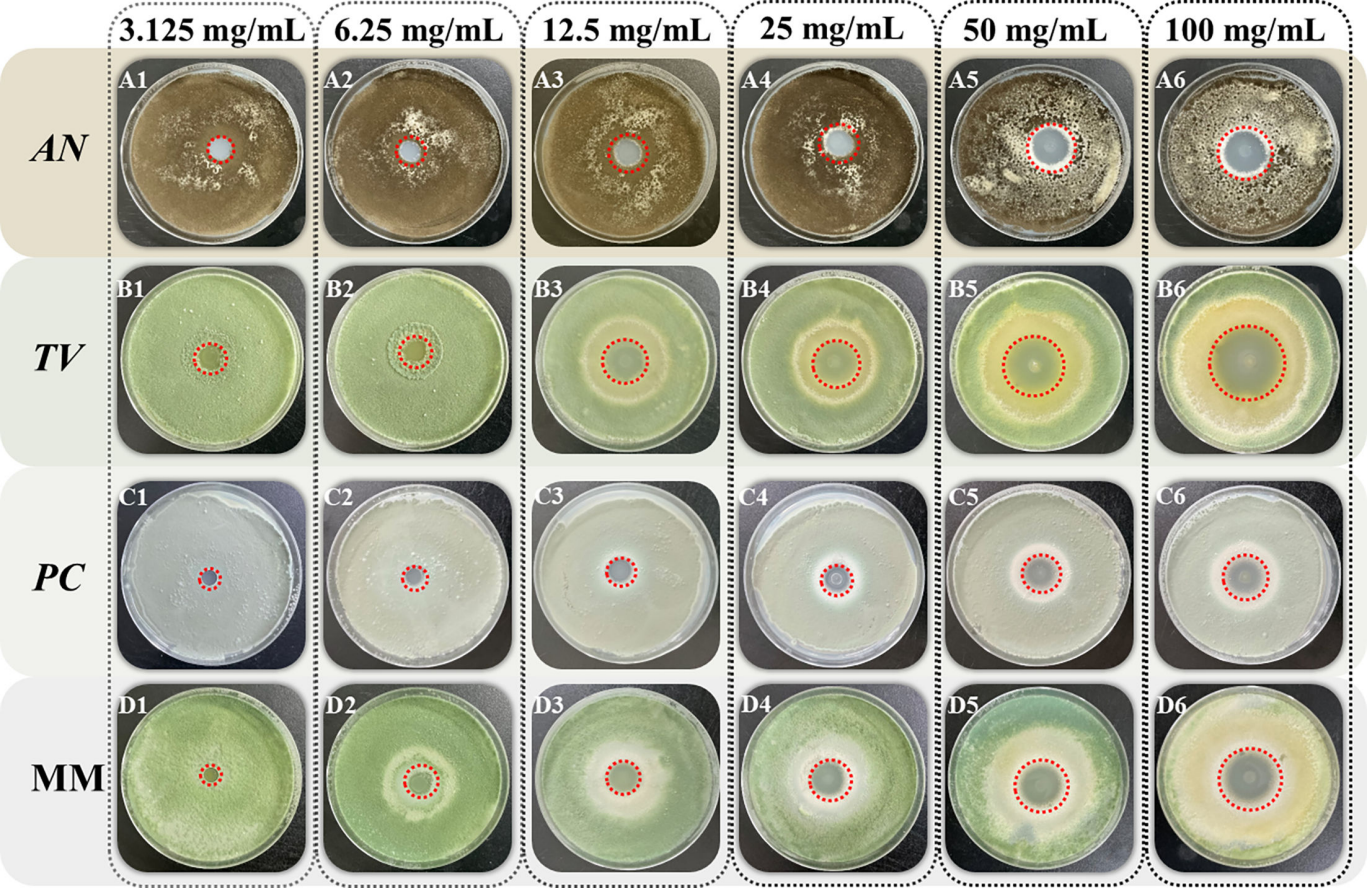
Inhibitory effect of oregano essential oil on the growth of bamboo mildew.

**Fig 4:**
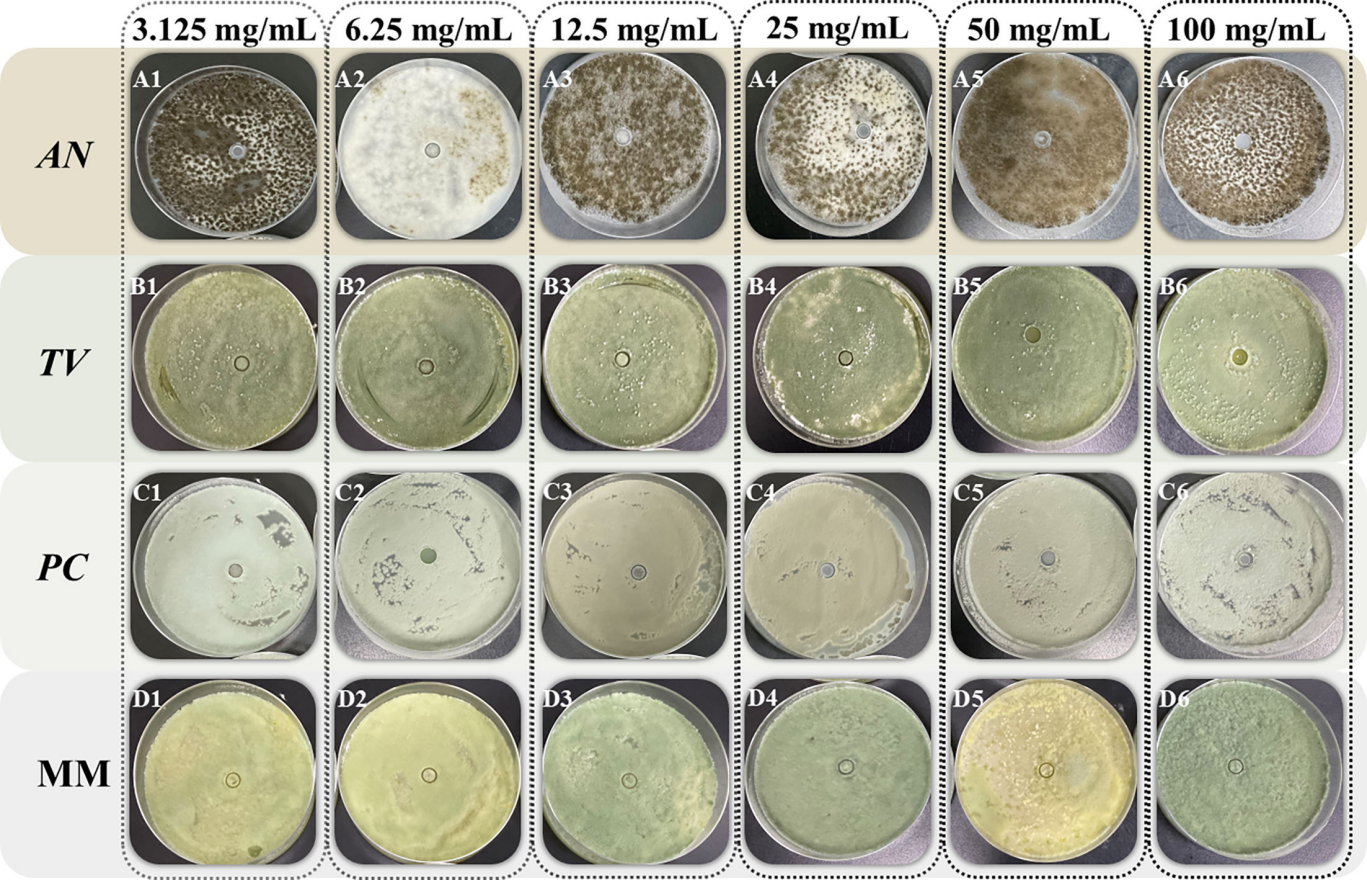
Inhibitory effect of fennel essential oil on the growth of bamboo mildew.

**Fig 5:**
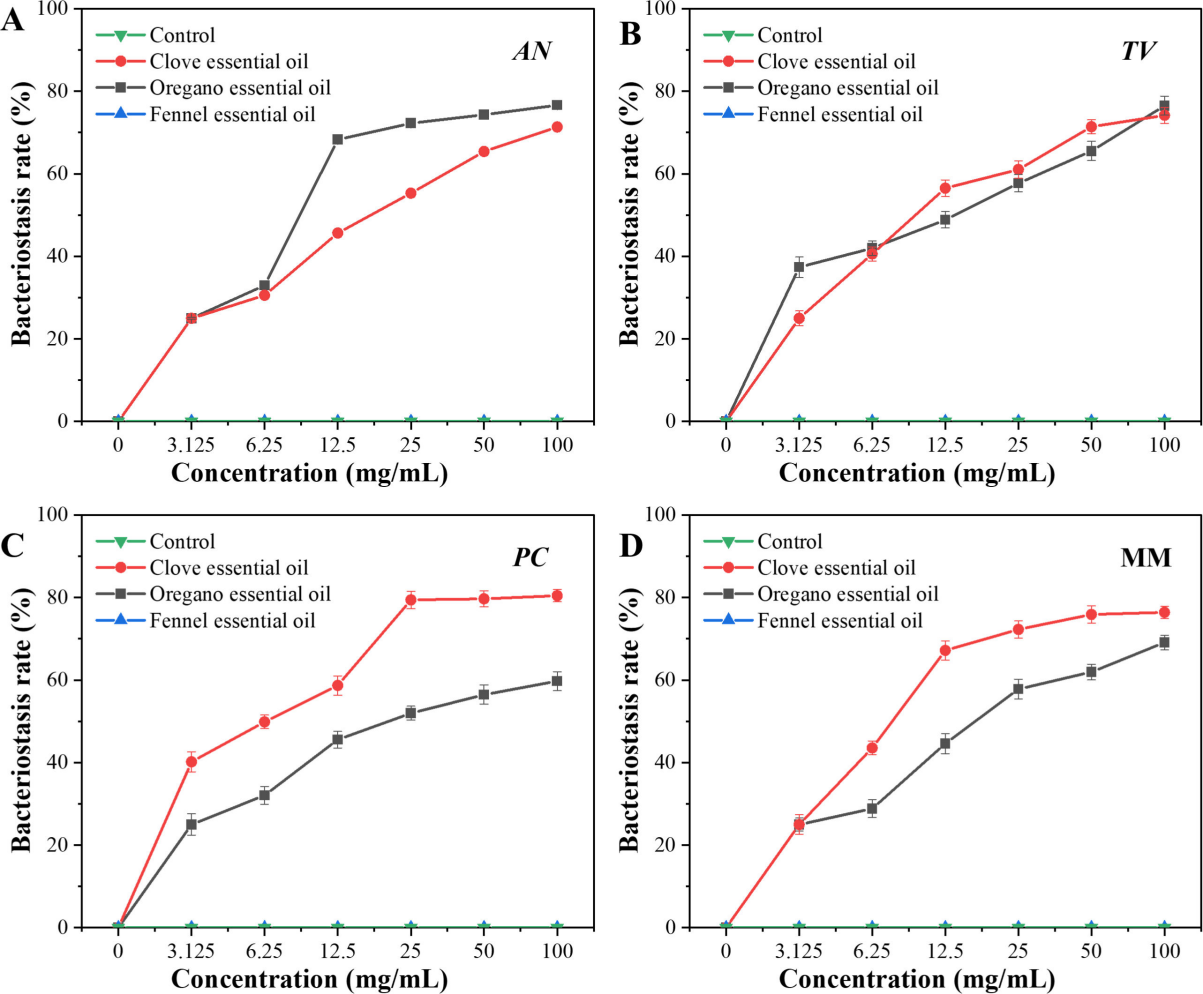
Effects of different concentrations of three spice essential oils on the fungistatic rate of bamboo mildew.

The Oxford Cup method was performed to investigate the effect of different concentrations of spice essential oil against AN, TV, PC, and MM based on the diameter of the fungistatic ring of bamboo mildew. The fungistatic rate was calculated based on the diameter of the fungistatic ring to characterize the anti-mildew ability of spice essential oil. The results of the fungistatic ring diameter are shown in Fig. 2 through 4, and the results of the Inhibition Zone and fungistatic rate are shown in Table 2; Fig. 5. The fungistatic ring diameter results of 0 mg/mL in Fig. 3 and 4 are shown in Fig. 2.

**TABLE 2:** Inhibition zone diameters of essential oils (clove, oregano, and fennel) at various concentrations against bamboo mildew

	Concentration (mg/mL)	Inhibition zone diameters (mm)			
		AN	TV	PC	MM
Clove essential	0.000	0.00	0.00	0.00	0.00
	3.125	8.00	8.00	10.03	8.00
	6.250	8.95	10.10	11.97	10.63
	12.500	18.92	13.80	14.52	18.28
	25.000	21.62	15.40	29.17	21.63
	50.000	23.37	20.98	29.53	24.90
	100.000	25.68	23.22	30.68	25.43
Oregano essential	0.000	0.00	0.00	0.00	0.00
	3.125	8.00	9.58	8.00	8.00
	6.250	8.64	10.34	8.83	8.43
	12.500	11.04	11.74	11.02	10.83
	25.000	13.42	14.18	12.49	14.22
	50.000	17.33	17.40	13.78	15.77
	100.000	20.93	25.52	14.90	19.41
Fennel essential	0.000	0.00	0.00	0.00	0.00
	3.125	0.00	0.00	0.00	0.00
	6.250	0.00	0.00	0.00	0.00
	12.500	0.00	0.00	0.00	0.00
	25.000	0.00	0.00	0.00	0.00
	50.000	0.00	0.00	0.00	0.00
	100.000	0.00	0.00	0.00	0.00

In Fig. 2 and 3, large Inhibition Zones were observed with clove and oregano essential oils, indicating strong inhibition against mildew. Fennel essential oil demonstrates minimal antimildew activity, with no inhibitory effect observed against three types of mildew. As the concentration of clove and oregano essential oils increased, the diameter of the inhibition zones also increased, demonstrating a positive correlation between the fungistatic effect and oil concentration. This phenomenon shows that the fungistatic effect is positively correlated with the concentration of essential oils. It can be seen from Table 2 that when the concentration of clove essential oil is 100 mg/mL, the diameters of bacteriostatic zones of AN, TV, PC*,* and MM are 25.68 mm, 23.22 mm, 30.68 mm, and 25.43 mm, respectively, all of which are >20 mm, indicating that clove essential oil is extremely sensitive to four kinds of mildew. Oregano essential oil exhibits extremely high sensitivity to *AN* and *TV*, with moderate sensitivity to *PC*. Fennel essential oil shows no sensitivity to any of the three mildews.

As can be seen from Fig. 5, clove essential oil has the best fungistatic effect against *AN* with a fungistatic rate of 76.64%. Meanwhile, oregano essential oil has a better fungistatic performance against *TV*, with a fungistatic rate of 76.49%.

### MIC and MFC values of spice essential oil against bamboo mildew

The MIC and MFC of spice essential oil against bamboo mildew were determined by multiple dilution methods (Table 3).

**TABLE 3:** MIC and MFC of the two spice essential oils on bamboo mildew

Spice essential oils	MIC (mg/mL)				MFC (mg/mL)			
	AN	TV	PC	MM	AN	TV	PC	MM
Clove essential oil	1.37	1.76	1.37	1.42	1.56	1.96	1.56	1.80
Oregano essential oil	2.44	1.95	3.42	4.88	5.37	2.20	3.91	5.87

MIC and MFC are crucial in mildew prevention experiments as they help determine the lowest effective concentrations of antimicrobial agents required to inhibit or kill fungi, ensuring effective mildew control measures.

Because fennel essential oil essentially lacks antibacterial activity, it has minimal inhibitory effects against three types of mold. Consequently, MIC and MFC experiments were not conducted. The MIC of clove essential oil against AN, TV, PC, and MM is small, especially for PC (Table 3), suggesting clove essential oil has the largest inhibitory effect on *PC* growth. Therefore, clove essential oil does not need to reach the maximum MIC of a single mildew to achieve the effect of inhibiting all three mildews simultaneously. Compared with the MIC value of clove essential oil against bamboo mildew, the values of oregano essential oil is slightly higher, indicating that the bacteriostasis of oregano essential oils is weaker than clove essential oil. Plant essential oil can inhibit the growth and reproduction of microorganisms owing to their specific active ingredients. Previous studies have shown that the antibacterial activity of essential oils is provided by their main compounds (16). Ju (17) showed that clove essential oil has different degrees of inhibition on some mildews, and the anti-mildew active component of clove essential oil is mainly eugenol. Carvacrol is the anti-mildew active ingredient of oregano essential oil (18). Based on the chemical composition analysis of spice essential oil, the content of eugenol, which is the main component of clove essential oil, is 87.12%. The main component of oregano essential oil is carvacrol which accounts for 50.72%. Therefore, the anti-mildew properties of spice essential oil are closely associated with their main anti-mildew components. Deans showed that most components have benzene rings and conjugated double bond structures, such as eugenol and citral, for active electron transport and interactions with ion channel proteins on the surface of the microbial cell membranes. This changes the permeability of the cell membrane and inhibits microbial growth (19). Based on the composition, plant essential oils, whose main components are rich in benzene rings and conjugated double bond structures, can exhibit more potent fungistatic activity (20). The MIC values against MM were greater than those of the remaining three strains of monobacte, probably due to the different mechanisms of action of the two essential oils on the mildews. The MFC values against *PC* were smaller with clove essential oil and smallest with oregano essential oil.

This indicated that different types of mildew have different tolerance against different essential oils. *AN* had the least tolerance and was most likely to be killed by clove essential. As the most active mildew in bamboo mildew, *AN* has less tolerance against essential oils, which can effectively eradicate bamboo mildew. In summary, the MIC and MFC values of clove essential oil against bamboo common mildew were lower than those of Oregano essential oil. Thus, a lower concentration of essential oil can achieve the effect of inhibiting or killing mildew. Clove essential oil has the best anti-mildew effect among the three essential oils.

### Effect of clove essential oil on mycelial morphology and cell microstructure of bamboo mildew

**Fig 6:**
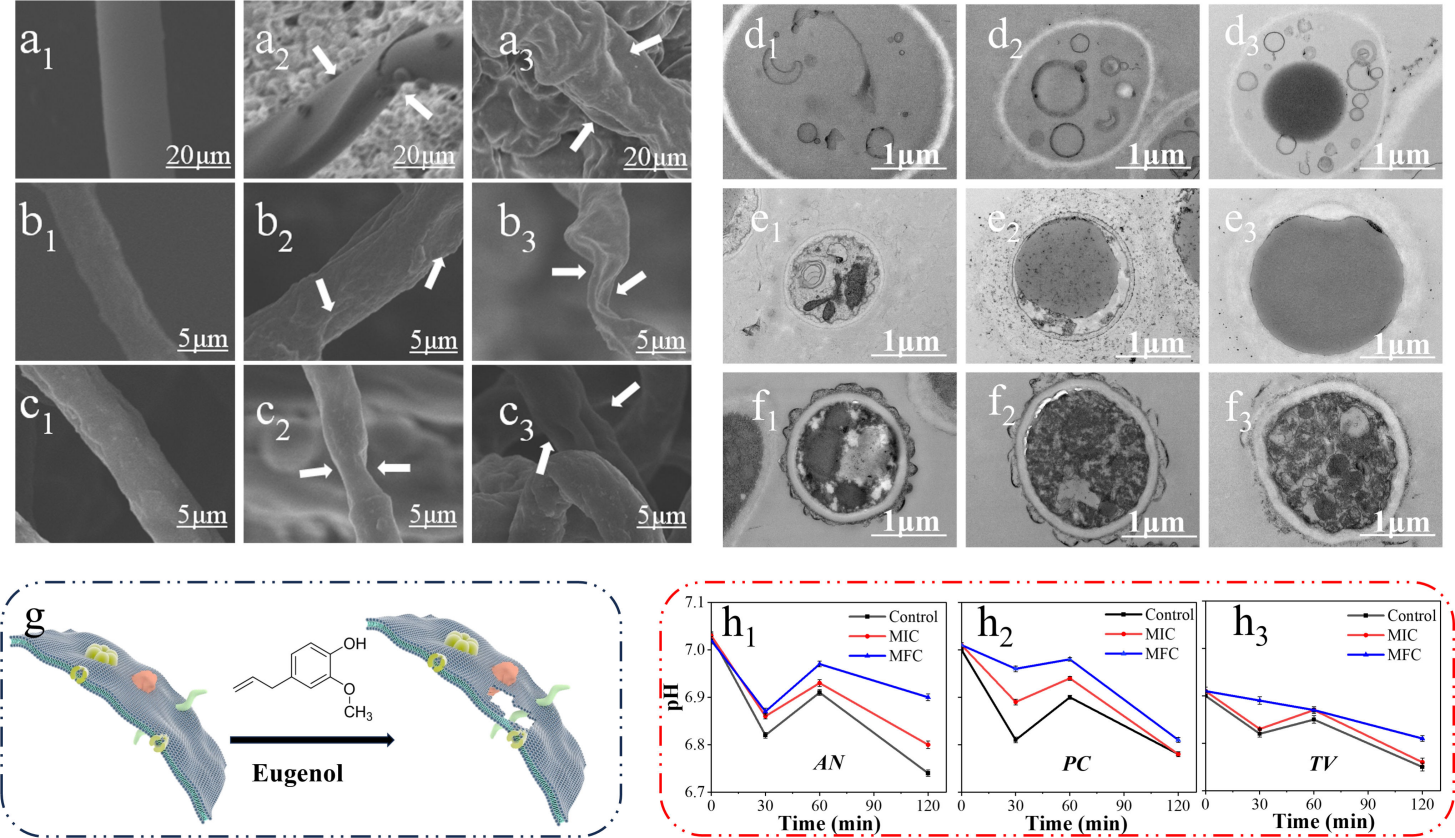
Effect of clove essential oil concentration on bamboo mildew mycelium morphology groups included *AN* control, MIC treatment, and MFC treatment (a_1_, a_2_, and a_3_), *TV* control, MIC treatment, and MFC treatment (b_1_, b_2_, and b_3_), and *PC* control, MIC treatment, and MFC treatment (c_1_, c_2_, and c_3_), respectively, The impact of clove essential oil concentration on the microscopic structure of bamboo mildew included *AN* control, MIC treatment, and MFC treatment (d_1_, d_2_, and d_3_), *TV* control, MIC treatment, and MFC treatment (e_1_, e_2_, and e_3_), and *PC* control, MIC treatment, and MFC treatment (f_1_, f_2_, and f_3_), respectively, (g) the potential antimildew mechanism of clove essential oil and (h) effect of clove essential oil on pH value of extracellular fluid of bamboo mildew.

Based on the results of the anti-mildew tests, it was found that clove essential oil exhibited the highest degree of anti-mildew activity among the tested essential oils. Therefore, it was deemed as the most suitable essential oil for further analysis in the study. The MIC and MFC of clove essential oil were utilized to treat bamboo mildew, followed by observation of the mycelium morphology of bamboo mildew using SEM. The resulting images are shown in Fig. 6a through c.

Figure a through c shows that the mycelia were full and regular in shape, thick and uniform, and intact in structure in the control group (a_1_**, b_1_, c_1_**). In the MIC-treated group, the mycelia of the mildew treated with *AN* (a_2_) was shriveled and showed ruffling and distorted shapes on the surface and partial fragmentation. The mycelia of the mildew treated with *TV* (b_2_) was also shriveled, and the surface was bumpy and uneven in thickness. The mycelia of the mildew treated with *PC* (c_2_) appeared to be shriveled, and the surface of the mycelia was bumpy and uneven. In the MFC-treated group, the mycelia of *AN-treated* (a_3_) mildews were severely shriveled and severely distorted in shape with a rough surface and overall irregular twisting. The mycelia of *TV-treated* (b_3_) mildew were severely shriveled, distorted in shape, and had a structure close to the broken state. The mycelia of *PC-treated* (c_3_) mildews were not only shriveled and rough but also appeared to be distorted.

Clove essential oil at different concentrations caused considerable damage to the mycelial shape and structural integrity of the mildew. Clove essential oil is a mixture of a large class of different components, which act on mildew in many ways. Therefore, its underlying fungistatic mechanism is more complicated. Eugenol disrupts the mildew cell membrane permeability, making intracellular substances, such as proteins, leak. Cytoplasmic aggregation affects gene expression related to cell growth and enables phage apoptosis (17, 19, 21). Some studies have shown that essential oil first destroys the cell membrane of microorganisms, resulting in different degrees of damage and deformed cell morphology on the surface of the cell membrane. The integrity of the cell membrane is critical to the survival of bacteria and is a key factor in maintaining the basic metabolism in cells. Destroying the integrity of the cell membrane can lead to microbial death (22). Clove essential oil can damage and destroy the cell membrane of bamboo mildew, causing serious shrinkage and distortion of mildew mycelium, rough surface with holes, or partial structural rupture, thereby inhibiting or killing mildew.

The MIC and MFC of clove essential oil were used to treat bamboo mildew, and TEM was used to observe the effect of clove essential oil on the cell microstructure of bamboo mildew, as shown in Fig. 6D through F.

In Fig. 6d through f, we can clearly see that the fungal hyphae of three types of mildews (d_1_, e_1_, f_1_) in the control group, which have intact cell structures and their internal structures maintain normal morphology and spatial position. The entire cell is round and plump, and the cell wall and membrane grow evenly, maintaining stability in the internal and external environment of the cell. In the MIC group, the thickness of the AN (d_2_) cell membrane became thinner and the cell wall became transparent. In TV (e_2_), there is partial dissolution of the cell wall, dissolution of cell contents, and loss to the outside of the cell, resulting in a large dark cavity. In PC (f_2_), the cell wall was also partially dissolved, the cell structure was disrupted and dissolved, and the whole cell was slightly deformed. In the MFC group, the cell wall and membrane thickness of AN (d_3_) further thinned, and even some cell membranes dissolved and disappeared; TV (e_3_) clearly shows that all cell contents have been lost to the outside of the cell; the cell wall of PC (f_3_) significantly dissolves and dissipates, further deforming the entire cell shape. It can be seen that the components in clove essential oil may dissolve the cell wall, destroy the cell membrane, cause the leakage of substances inside the cell, and cause cell death.

As shown in Fig. 6g, this represents the potential antimildew mechanism of clove essential oil. The integrity of cell structure is crucial for cell survival. The structural integrity of cells means that all components of cells (such as cell membrane, nucleus, mitochondria, endoplasmic reticulum, etc.) maintain correct morphological and positional relationships and can interact and transmit information normally. When the cell structure is damaged or destroyed, it not only affects its normal physiological activities but also causes cell death. For example, the rupture of a cell membrane can cause ion permeation within the cell, molecular loss within proteins, and disruption of intracellular stability.

### Effect of clove essential oil on the pH of the extracellular fluid of bamboo mildew cell

A micro pH meter was used to detect the changes in the extracellular pH of bamboo mildew after being treated with different concentrations of clove essential oil at different times. It was used to determine the damage of clove essential oil on the cell wall of bamboo mildew (Fig. 6h).

Membrane damage is considered a hallmark of biotic and abiotic stress responses. The cell membrane determines the ability of the cell to withstand alterations in environmental conditions and can, therefore, be used as an indicator to assess cell damage or death (23). In normal cells, the extracellular fluid pH is essentially neutral, and the intracellular fluid pH is slightly acidic. Therefore, when the extracellular fluid is slightly acidic, maintaining the balance of intracellular and external fluid exocrine acid for the cell will decrease the discharge of intracellular protons. As shown in Fig. 6h, it can be observed that during the initial 0–30 min period, the pH of bamboo extracellular fluid decreased, with a slighter decrease in the treatment group compared with that in the control group. This indicated that clove essential oil damages the cell wall and reduces its biological activity, leading to acid secretion by the cells and subsequent reduction in the pH of extracellular fluid. However, some degree of cell damage in the treatment group resulted in a lower amount of proton outflow, thereby slowing down the decrease in pH value. During the 30–60 min treatment period, the pH value of extracellular fluid increased in both the control and treatment groups. This was due to the self-regulatory ability of the cells, where they began to produce acid in response to a lower extracellular pH value relative to intracellular pH. This increased the pH value of the extracellular fluid. After 60 min of treatment, the pH of the extracellular fluid decreased again, which was attributed to the prolonged exposure of the mildew to external stimuli, leading to damage in the permeability of the cell membrane and subsequent proton and potassium ion leakage (24, 25). Such leakage decreased the pH gradient across the cell plasma membrane, ultimately resulting in cell death and an anti-mildew effect (26, 27). These findings suggested that clove essential oil disrupted the bioactivity of the mildew cell wall, leading to an imbalance in intracellular and extracellular pH, disrupting cell wall permeability, and causing massive proton leakage, ultimately achieving the inhibition or killing effect of mildew.

### Appearance quality of clove essential oil mildew proof sliced veneer plybamboo

**Fig 7:**
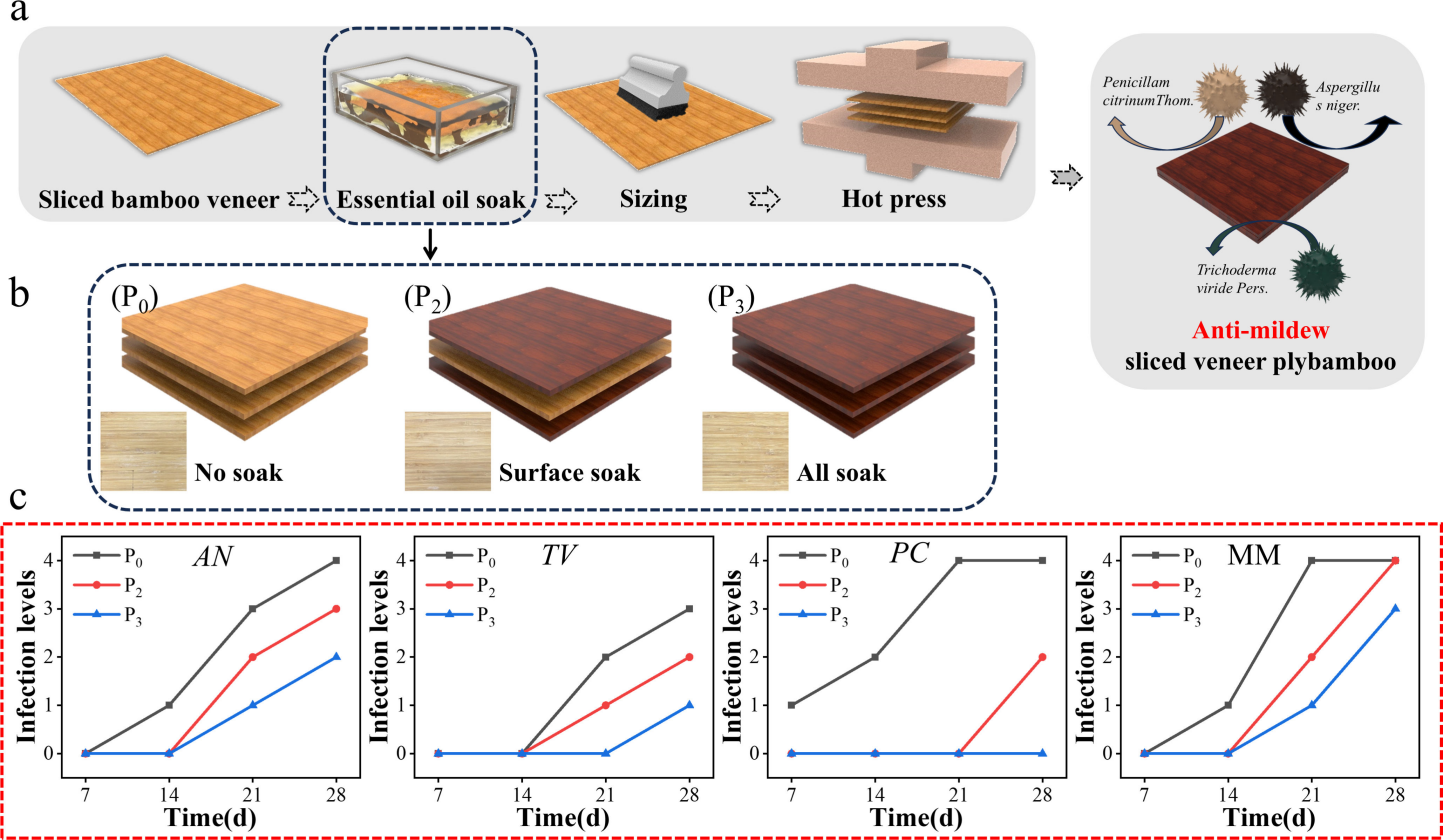
(a) Finished products of sliced veneer plybamboo with different paring methods (Figure P_0_: sliced veneer plybamboo in the control group; Figure P_2_: non-impregnated core layer sliced veneer plybamboo; Figure P_3_: impregnated all layers sliced veneer plybamboo, (b) the manufacturing process of sliced veneer plybamboo, and (c) growth grading values of AN, TV, PC*,* and MM after 28 days of sliced veneer plybamboo with different formation methods.

The manufacturing process of sliced veneer plybamboo, as shown in Fig. 7a, involves immersing thin bamboo sheets in an anti-mildew solution, applying adhesive, and finally subjecting them to heat and pressure to produce sliced veneer plybamboo.Digital photo images of the appearance of three types of sliced veneer plybamboo (P_0_, P_2_, P_3_) were shown in Fig. 7b. Through visual inspection and measurement tools, the results are checked one by one, as shown in Tables 4 and 5.

**TABLE 4:** Appearance quality of sliced veneer plybamboo by different formation methods^*a*^

Inspection items	Appearance quality		
	P_0_	P_2_	P_3_
Lamination	—	—	—
Surface blistering	—	—	—
Overpenetration	Slight	Slight	—
Plate misalignment	—	—	—
Dehiscence	—	—	—
ther defects	—	—	—

^*a*^ "—" indicates that there are no corresponding cases present for that specific entry.

**TABLE 5:** Effect of different prefabrication methods on physical and chemical properties of sliced veneer plybamboo

Classification	Bonding performance (MPa）	Wood failure (%）	Moisture content (%）
P_0_	0.81	85%	9.25
P_2_	0.80	70%	9.63
P_3_	0.75	55%	9.77

Physicochemical properties of clove essential oil mildew proof sliced veneer plybamboo. The physical and chemical properties of sliced veneer plybamboo prepared by three different methods are shown in Table 5.

The bonding strength of P_2_ and P_3_ decreased slightly compared to P_0_ because volatile components from clove essential oil formed a film on the veneer surface during impregnation. This hindered the physical and chemical combination between the bamboo veneer and adhesive. Additionally, reactions with carboxyl-containing compounds in the bamboo veneer led to esterification products, reducing adhesive penetration and strength. Moreover, the oiliness and poor wetting performance of clove essential oil hindered effective adhesive wetting and firm veneer combination.

### Mildew-proof properties of clove essential oil sliced veneer plybamboo

**Fig 8:**
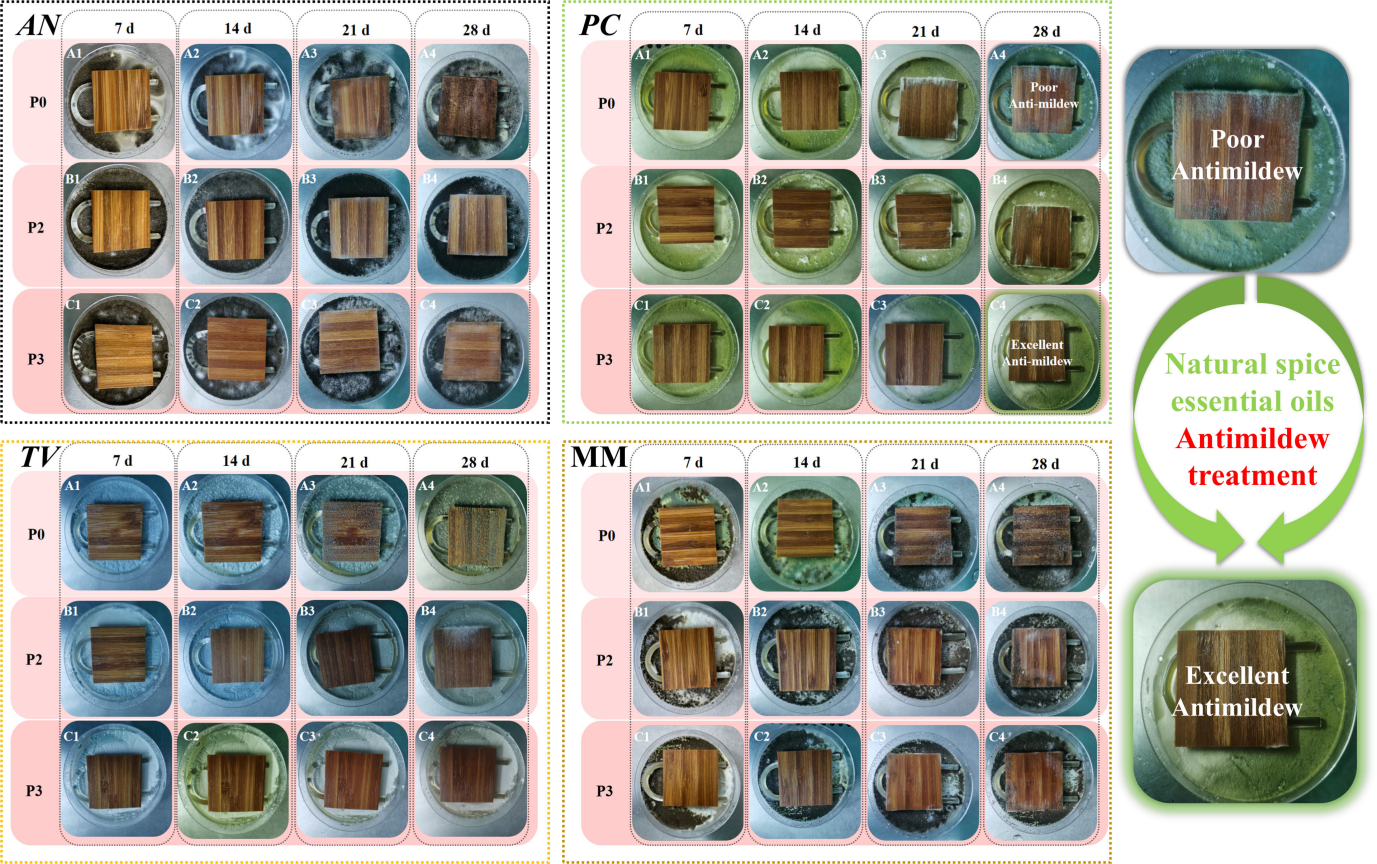
Effect of different paring methods on the mildew prevention of AN, TV, PC, and MM by sliced veneer plybamboo for 7–28 days.

The mildew resistances of three types of sliced veneer plybamboo are shown in Fig. 8. The data presented in Fig. 8 clearly demonstrate the effectiveness of clove essential oil in inhibiting mildew growth in thinly sliced bamboo. Plywood treated with three layers of clove essential oil under excessive pressure (P_3_) exhibited the highest resistance to mildew, with a lower grade value for AN growth compared to other treatment groups. Similarly, Fig. 8 illustrates the significant inhibitory effect of clove essential oil on TV growth, with P_3_ showing the lowest mildew growth grading value on the 28th day. Moreover, clove essential oil effectively suppresses PC growth, as evidenced by the absence of PC infection in P_3_ compared to P_0_ and P_2_. These findings collectively suggest that clove essential oil offers a natural and effective solution for mitigating mildew contamination in thinly sliced bamboo.

In all, clove essential oil anti-mildew treatment of sliced veneer plybamboo belongs to wood-based panels with anti-mildew quality grade 1. Therefore, clove essential oil may be an effective anti-mildew agent for preventing mildew growth and contamination in sliced veneer plybamboo. The use of this anti-mildew agent may be an environmentally friendly and cost-effective method that can improve the quality and lifespan of bamboo products.

### Conclusion

This study concludes that clove essential oil is a highly effective antifungal agent for protecting bamboo from mildew and fungal infestations. Results showed that clove essential oil exhibited significant inhibitory effects, with minimum inhibitory concentrations (MICs) ranging from 1.37 to 1.42 mg/mL, and minimum fungicidal concentrations (MFCs) ranging from 1.56 to 1.80 mg/mL against various fungal strains. Its application not only successfully inhibits the growth of harmful fungi but also preserves the structural integrity and appearance of bamboo products, aligning with industry standards. The findings suggest that clove essential oil could be a valuable and sustainable alternative for fungal control in bamboo processing and preservation, offering a practical solution that does not compromise the material’s quality or usability. This positions clove essential oil as a promising candidate for broader use in natural antifungal strategies within the bamboo industry.

## AFTER EPUB

This article was published on 10 October 2024 with an error in the affiliations. The affiliations were corrected in the current version, posted on 21 October 2024.
